# Enhanced antibacterial and antioxidant capabilities using indole-modified 1-phenyl-1*H*-pyrazolo[3,4-*b*]pyridine-5-carbonitrile derivatives, molecular docking evaluation and *in silico* ADMET prediction

**DOI:** 10.1039/d5ra07372c

**Published:** 2025-12-01

**Authors:** Hemat S. Khalaf, Ahmed F. El-Sayed, Ahmed H. Shamroukh

**Affiliations:** a Photochemistry Department, Chemical Industries Research Institute, National Research Centre 33 El Buhouth Street, P. O. Box 12622 Cairo Egypt hematsalama2016@gmail.com ahshamroukh@yahoo.com; b Microbial Genetics Department, Biotechnology Research Institute, National Research Centre Giza Egypt; c Egypt Center for Research and Regenerative Medicine (ECRRM) Cairo Egypt

## Abstract

A new series of 1-phenyl-1*H*-pyrazolo[3,4-*b*]pyridine-5-carbonitriles 4a–f, were prepared *via* a Knoevenagel condensation reaction followed by Michael addition for their antimicrobial and antioxidant properties, through the one-pot three-component reaction of 3-indolyl-3-oxopropanenitrile (1), aromatic aldehydes 2 and 1*H*-pyrazol-5-amines 3 in ethanol in the presence of triethylamine as a catalyst, which upon cyclization and auto-oxidation yielded the corresponding product in excellent proportion. Elemental analysis and spectroscopic methods were used to investigate the novel derivatives. The newly synthesized compounds' antibacterial and antioxidant properties were assessed, and their interactions with important proteins were investigated using molecular docking. Strong action was demonstrated by compounds 4a, 4c, and 4f against the bacterial strains *P. aeruginosa*, *K. pneumoniae*, *S. aureus*, and *E. coli*. DPPH radical scavenging techniques were used to evaluate their antioxidant capacities, demonstrating their capacity to counteract oxidative stress. Molecular docking and ADMET/drug-likeness screening revealed favorable binding energies and adherence to Lipinski's rules, indicating these compounds could be promising orally bioavailable drug candidates.

## Abstract 2

A series of 1-phenyl-1*H*-pyrazolo[3,4-*b*]pyridine-5-carbonitriles were synthesized and characterized. The compounds showed notable antimicrobial activity against major bacterial strains and exhibited antioxidant potential in the DPPH assay.

## Graphical abstract

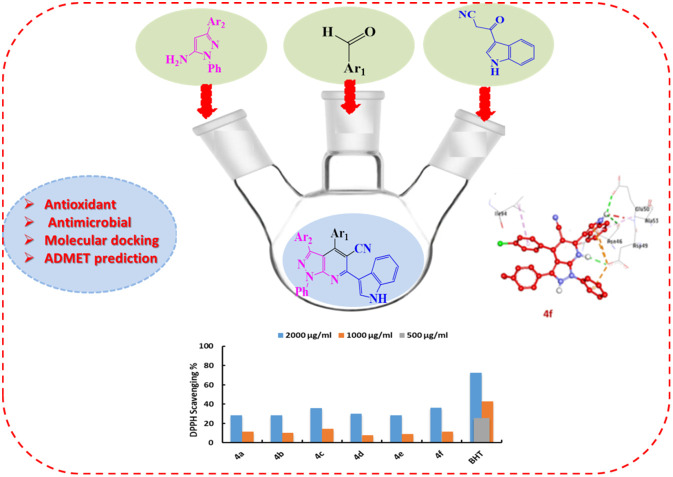

## Introduction

Infectious diseases are a major cause of death in developing and developed countries.^1,2^ These diseases are treated with antibiotics. The discovery of penicillin in 1928 and its use as an antibiotic since 1938 have improved human health.^3^ However, the overuse of antibiotics, generally without prescription, has led to the emergence and spread of advanced strains of microbes that are resistant to the effectiveness of antibiotics.^4^ Several studies have implicated antimicrobial resistance as a major threat to public health,^5,6^ Antimicrobial resistance makes antibiotic treatment ineffective, leading to increased treatment costs and higher rates of morbidity and mortality, especially in immunocompromised patients.^7^ Therefore, there is a need to develop new antibacterial compounds with novel targets and selective toxicity to overcome this problem.^7,8^ Free radicals (FR) are produced in the body, but the body cannot expel them *via* endogenous or exogenous antioxidants, resulting in oxidative stress.^9^ Throughout various biochemical pathways, multiple reactions take place in which the boosters are the reactive oxygen species (ROS), such as hydrogen peroxide (H_2_O_2_) and the superoxide radical anion (O_2_˙^−^), among others. Increased quantities of FR may result in damage of biomolecules, leading to severe pathological diseases such as atherosclerosis, cancer, diabetes, cardiovascular, and chronic inflammation.^10^ To inactivate the excess ROS, biological systems contain endogenous antioxidant mechanisms, including superoxide dismutase (SOD), catalase (CAT), glutathione peroxidase, as well as non-enzymatic compounds like bilirubin and albumin. Thus, the consumption of antioxidants is the most efficient method to avoid many diseases related to the production of high levels (ROS).^11^ The clinical administration of drugs and the chemoprophylaxis of different diseases that happen *via* oxidizing agents need the development of new antioxidants that have the predicted antioxidant activity and desired pharmacological properties.^12^

**Fig. 1:**
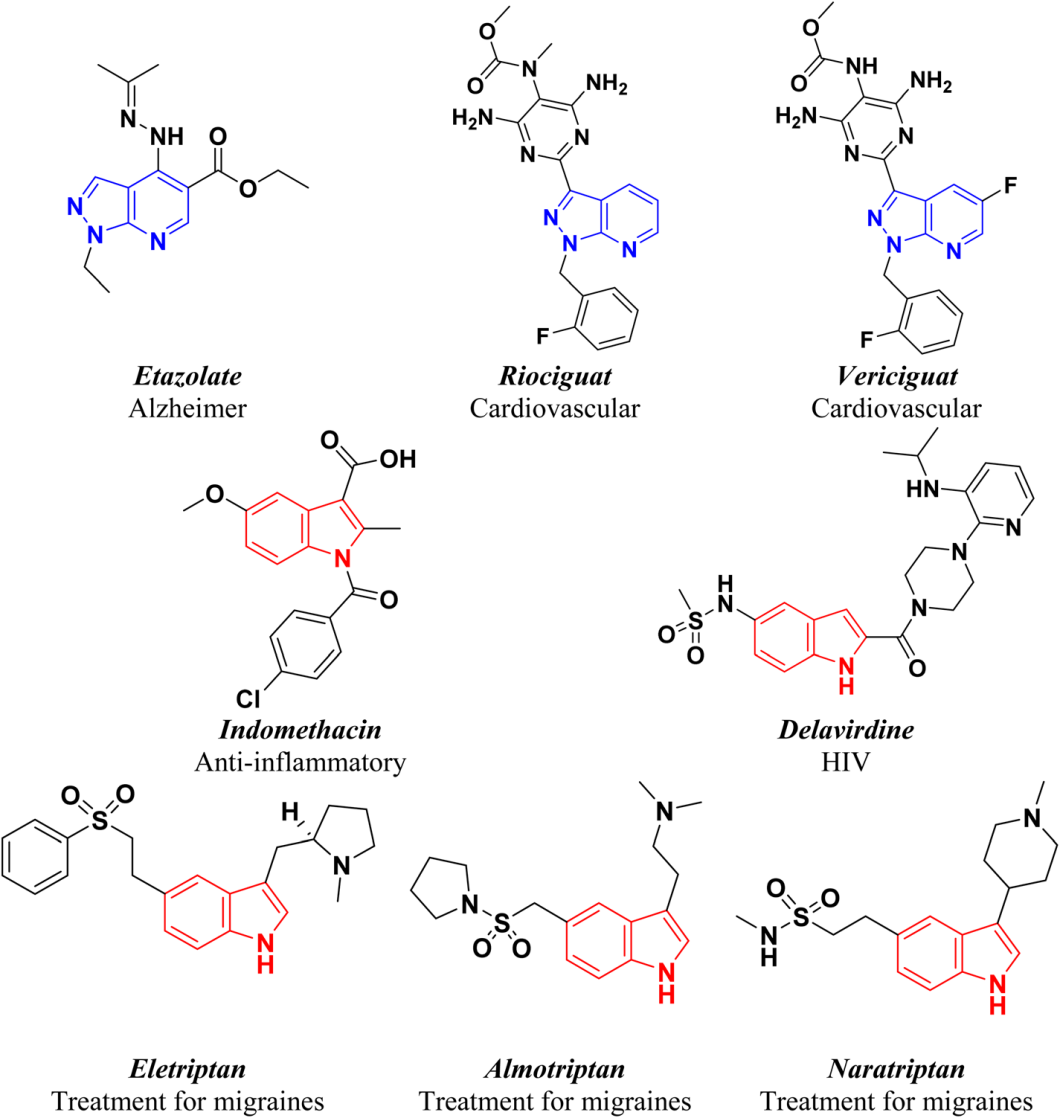
Chemical structures of some clinical drugs having pyrazolo[3,4-*b*]pyridine and indole moieties.

Pyrazolo[3,4-*b*]pyridines, as one of the classes of nitrogen-containing heterocycles, can form a variety of interactions with the active centers of cell components making these compounds useful as anticancer,^13–15^ antibacterial,^16^ antimalarial,^17^ antiviral,^18^ anti-inflammatory,^19^ antitrypanosomal,^20^ anti-hypertension and pulmonary hypertension,^21,22^ anti-Alzheimer's agents.^23^ Also, some clinical drugs contain pyrazolo[3,4-*b*]pyridine moieties are Etazolate for the treatment of Alzheimer's disease, Riociguat is used to treat two forms of pulmonary hypertension (PH): chronic thromboembolic pulmonary hypertension (CTEPH) and pulmonary arterial hypertension (PAH). Vericiguat is a medication used to reduce the risk of cardiovascular death (Fig. 1).^24,25^

On the other hand, indole is a nitrogen-containing heterocyclic compound considered a model for the design of drugs such as the classical non-steroidal anti-inflammatory drugs (NSAIDs) Indomethacin,^26^ Delavirdine is a non-nucleoside reverse transcriptase inhibitor used to treat HIV infection.^27^ Eletriptan, Almotriptan and Naratriptan are 5-HT1B/1D receptor agonists used to treat migraines (Fig. 1).^28^

Additionally, indole derivatives have been shown to possess a variety of biological properties, including antitumor,^29,30^ antifungal,^31^ analgesic,^32^ anti-inflammatory,^33^ antipyretic,^34^ anticonvulsant,^35^ and selective COX-2 (cyclooxygenase-2) inhibitory properties.^36^ Antioxidant properties are found in various indole derivatives.^37,38^ Melatonin derivatives have proved particularly useful^39^ due to their potent capacity to scavenge reactive oxygen species (ROS) and reactive nitrogen species (RNS).^40^

In addition, molecular docking is widely utilized to predict how small therapeutic compounds interact with their protein targets, providing valuable insights into the molecules' affinity and activity. In pharmaceutical design, this process is essential. Given docking studies' biological and pharmacological importance, significant efforts have been made to improve algorithms for accurate docking predictions.^41^ Docking is vital for assessing interactions between synthesized compounds and protein receptors, offering essential information on their binding modes and potential biological properties.^42–44^

**Fig. 2:**
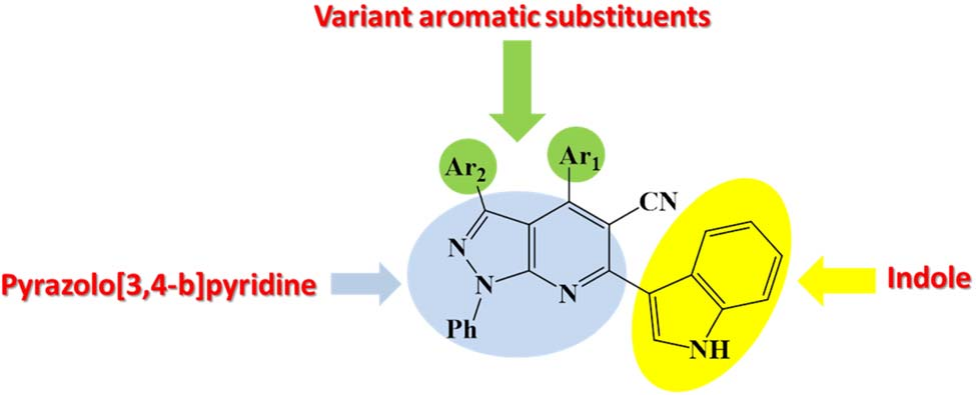
A strategy for designing (indole-pyrazolo[3,4-*b*]pyridine) hybrid.

Pyrazolo[3,4-*b*]pyridine and indole scaffolds are well-recognized for their diverse and potent biological activities, including antimicrobial, antioxidant, and anticancer properties. Motivated by the potential for synergistic interactions, we sought to integrate these two pharmacologically privileged moieties into a single molecular architecture to enhance their overall bioactivity. As part of our continued efforts to develop novel heterocyclic compounds with therapeutic potential,^45–48^ the present study reports the design and synthesis of a new series of 1-phenyl-1*H*-pyrazolo[3,4-*b*]pyridine-5-carbonitrile derivatives functionalized with an indole unit. The synthesized compounds were subsequently evaluated for their antimicrobial and antioxidant activities, aiming to identify candidates with promising dual-function bioactivity (Fig. 2).

## Results and discussion

### Chemistry

**Scheme 1:**
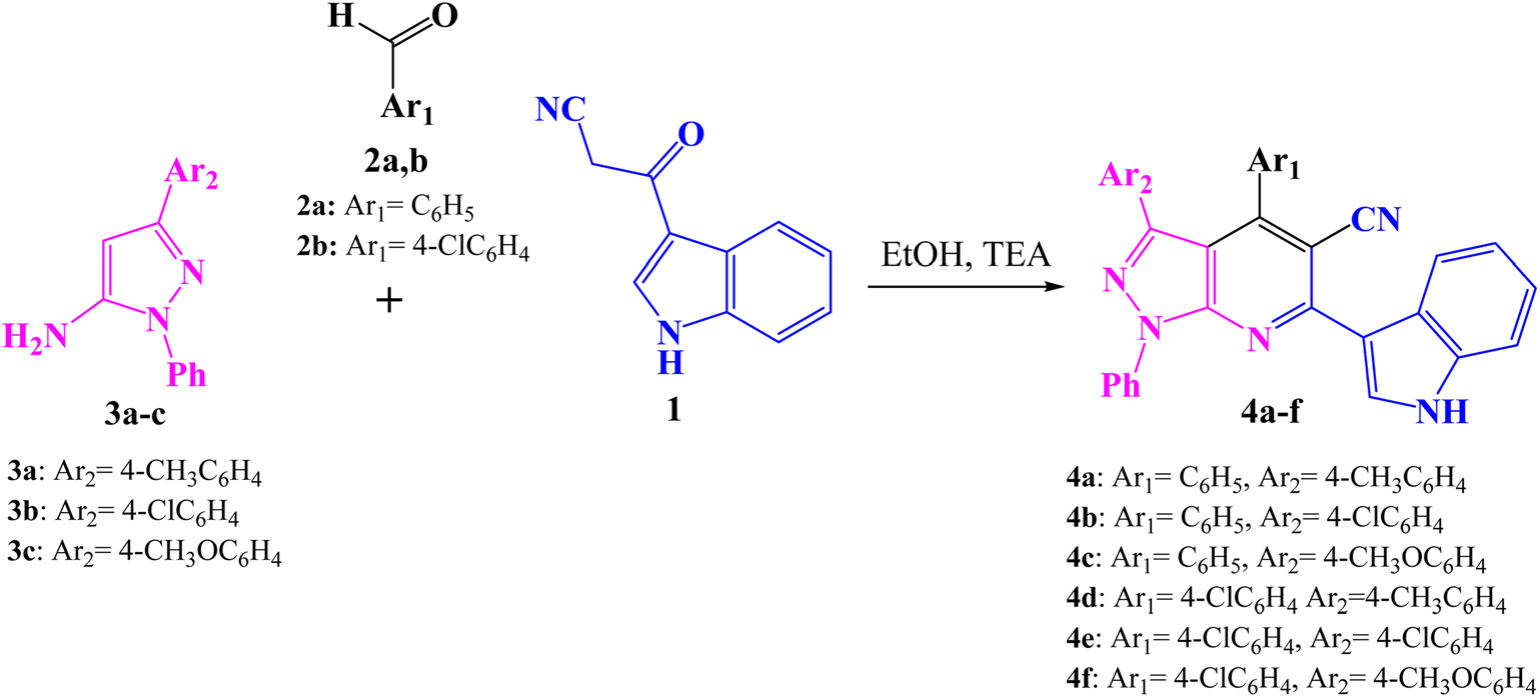
Synthetic pathway of 1-phenyl-1*H*-pyrazolo[3,4-*b*]pyridine-5-carbonitrile derivatives 4a–f.

**Scheme 2:**
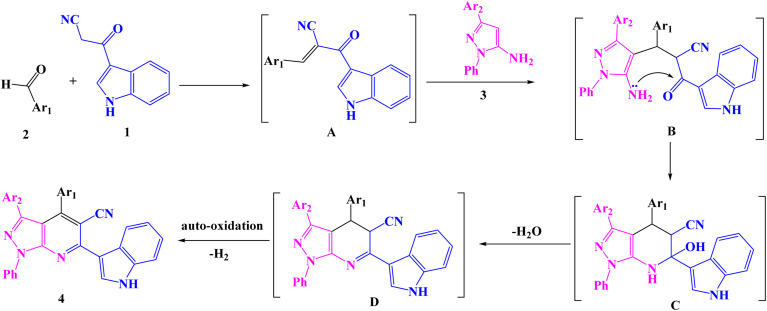
The proposed mechanism of the synthesizing pyrazolo[3,4-*b*]pyridine derivatives.

A series of 1-phenyl-1*H*-pyrazolo[3,4-*b*]pyridine-5-carbonitrile derivatives 4a–f were prepared *via* a one-pot, three-component reaction of 3-indolyl-3-oxopropanenitrile (1), aromatic aldehydes 2a–b and 1*H*-pyrazol-5-amines 3a–c in ethanol in the presence of triethylamine (TEA) as a catalyst (Scheme 1). The formation of product 4 could be explained firstly through the nucleophilic addition of 3-indolyl-3-oxopropanenitrile 1 to aldehyde 2 initiated by TEA to form the intermediate A (Knoevenagel product)^49^ Then adduct A underwent a Michael-type addition reaction with 1*H*-pyrazol-5-amine 3 to yield an adduct B intermediate. After that, intermediate B underwent intramolecular cyclization to give the intermediates C, which upon loss of water formed intermediate D. Finally by autoxidation of intermediate D produced the target compound 4 (Scheme 2).

The structure of the target compounds 4a–f was characterized using the IR spectra, ^1^H and ^13^C NMR spectroscopic data, and the elemental analysis (*cf.* Experimental). The IR spectra showed absorption bands at *ν* 3370–3380 cm^−1^ characterized for NH group of indoles, bands of aromatic moieties in their respective regions at *ν* 3045–3082 cm^−1^ and bands at *ν* 2217–2226 cm^−1^ for C≡N group, all established the desired structures (SI, S1 and S4). Meanwhile, the ^1^H NMR spectra showed singles at *δ* 11.64–11.94 ppm attributed to the NH groups (D_2_O exchangeable), besides the remaining protons of aromatic rings resonated at their usual chemical shift (*cf.* SI, S2, S5, S6, and S8). Also, the ^13^C NMR spectra showed singles attributed to methyl and methoxy groups, besides the remaining carbons of aromatic rings resonated at their usual chemical shift (SI, S7, S9 and S10). In addition, the mass spectra of compounds 4a–f gave molecular ion peaks confirming the synthesized compounds' structures (*cf.* SI, S3).

### Biological assays

#### Antioxidant activities of compounds

**Fig. 3:**
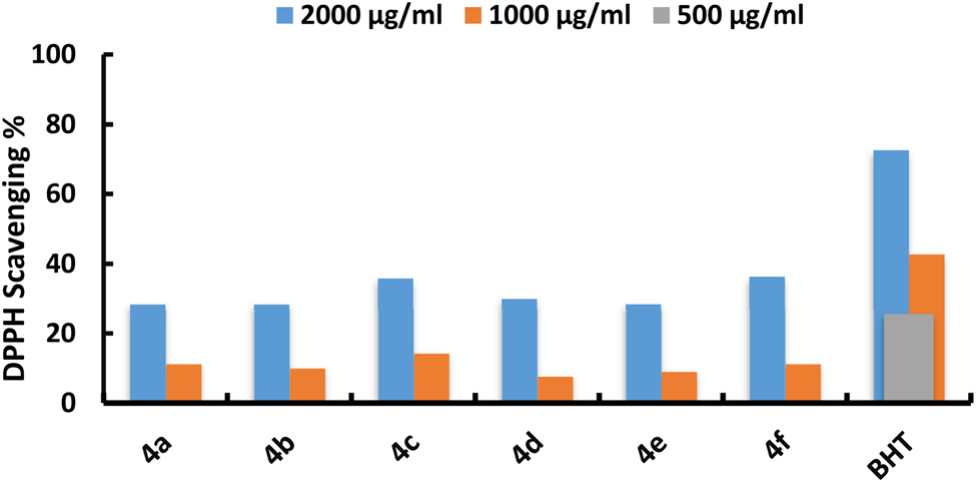
Antioxidant activity of synthesized compounds using DPPH method.

The prepared compounds were tested for antioxidant capability using DPPH and compared to the positive control, Butylated hydroxytoluene (BHT) at a concentration of 50 µg mL^−1^. Compounds 4c and 4f showed significant DPPH activity, with values of 35.80 ± 0.40, 14.22 ± 0.10, and 0.0, and 36.80 ± 0.40, 13.70 ± 0.12, and 0.0 at concentrations of 2.0, 1.0, and 0.5 mg mL^−1^, respectively. Moreover, compounds 4a, 4b, 4d, and 4e exhibited escalating DPPH activity from 11.22 ± 0.30, 9.98 ± 0.13, 7.58 ± 0.09, and 11.20 ± 0.11 at 1.0 mg mL^−1^ to 28.30 ± 0.30, 28.35 ± 0.18, 29.90 ± 2.8, and 28.30 ± 0.21 at 2.0 mg mL^−1^, respectively (Fig. 3). These compounds exhibit redox characteristics that allow them to act as reducers, hydrogen atom suppliers, and free radical scavengers, all of which contribute to their antioxidant activity. These compounds exhibit antioxidant capabilities by partnering the DPPH radical with a hydrogen atom or contributing electrons. Our results are consistent with the study by Khalaf*et al.*,^50^ which similarly assessed the antioxidant capabilities of synthesized compounds using DPPH scavenging tests. Furthermore, Jawhari *et al.*,^42^ shown that the antioxidant efficacy of produced compounds is related to the substituents present on the phenyl ring, emphasizing the favorable role of hydroxyl groups in improving antioxidant performance Table 1.

**Table 1:** Antioxidant activity of synthesized compounds

Code	Antioxidant activity DPPH%			IC50 mg mL^−1^
	2.0 mg mL^−1^	1.0 mg mL^−1^	0.5 mg mL^−1^	
Control (BHT)	72.55 ± 0.30	42.68 ± 0.70	25.60 ± 0.90	—
4a	28.30 ± 0.30	11.22 ± 0.30	0.0	3.533
4b	28.35 ± 0.18	9.98 ± 0.13	0.0	3.527
4c	35.80 ± 0.40	14.22 ± 0.10	0.0	2.793
4d	29.90 ± 2.8	7.58 ± 0.09	0.0	3.448
4e	28.30 ± 0.21	11.20 ± 0.11	0.0	3.533
4f	36.80 ± 0.40	13.70 ± 0.12	0.0	2.614

#### Antimicrobial activities of compounds against pathogenic microbial strains

All produced compounds were evaluated for antibacterial activity against a variety of bacterial species. The antimicrobial activity was examined across six different microorganisms, and the compounds 4a, 4c, and 4f shown action against all tested bacterial strains, resulting in considerable inhibition zones with diameters of 12.00 ± 0.10, 7.0 ± 0.10, and 6.0 ± 0.0 mm for *K. pneumoniae* ATCC25915, 14.20 ± 0.05, 4.5 ± 0.00, and 3.50 ± 0.0 mm for *S. aureus* ATCC25923, 10.0 ± 0.11, 8.0 ± 0.40, and 7.80 ± 0.60 mm for *K. pneumoniae* ATCC29212, and 11.0 ± 0.09, 8.5 ± 0.00, and 9.80 ± 0.00 mm for *P. aeruginosa* ATCC10145. Additionally, compounds 4b, and 4e only showed activity against *S. aureus* ATCC25923 with inhibition zones of 2.50 ± 0.10, and 6.50 ± 0.0, respectively. Notably, the yeast strain *C. albicans* did not show any inhibition activity with all compounds (Table 2). The most active compounds were further assessed for their antimicrobial activities to determine the minimum inhibitory concentration (MIC) values of compounds 4a, 4c, and 4f against pathogenic bacteria, as outlined in Table 3. Compound 4a displayed MIC values of 1.0, 1.0, 2.0, and 1.0 mg mL^−1^ against *E. coli* ATCC25915, *S. aureus* ATCC25923, *K. pneumoniae* ATCC29212, and *P. aeruginosa* ATCC10145, respectively. Compounds 4f showed MIC values of 2.0, 2.0, 2.0, and 1.0 mg mL^−1^ against the same bacterial strains. Compound 4c demonstrated MIC values of 1.0 mg mL^−1^ against all pathogenic bacteria, including *E. coli* ATCC25915, *S. aureus* ATCC25923, *K. pneumoniae* ATCC29212 and *P. aeruginosa* ATCC10145, respectively (Table 3).

**Table 2:** Antibacterial and antifungal activities of compounds on yeast and bacterial strains^a^

Compounds (2 mg mL^−1^)	Inhibition zone diameters (IZDs) (mm)				
	*C. albicans*	*E.coli* ATCC25915	*S. aureus* ATCC25923	*K. pneumoniae* ATCC29212	*P. aeruginosa* ATCC10145
4a	(−)	(+) 12.00 ± 0.10	(+) 14.20 ± 0.05	(+) 10.0 ± 0.11	(+) 11.0 ± 0.09
4b	(−)	(−)	(+) 2.50 ± 0.10	(−)	(−)
4c	(−)	(+) 7.0 ± 0.10	(+) 4.5 ± 0.00	(+) 8.0 ± 0.40	(+) 8.5 ± 0.00
4d	(−)	(−)	(−)	(−)	(−)
4e	(−)	(−)	(+) 6.50 ± 0.00	(−)	(−)
4f	(−)	(+) 6.0 ± 0.0	(+) 3.50 ± 0.00	(+) 7.80 ± 0.60	(+) 9.80 ± 0.00
Ciprofloxacin (50 µg mL^−1^)	—	(+) 11.00 ± 0.10	(+) 12.00 ± 0.11	(+) 8.20 ± 0.08	(+) 14.32 ± 0.54

a Values are given as mean ± standard error.

**Table 3:** The minimum inhibitory concentration (MIC) values of compounds 4a, 4c, and 4f against pathogenic bacteria^a^

Compounds	Concentration mg mL^−1^		Inhibition zone diameters (IZDs) (mm)		
		*E. coli* ATCC25915	*S. aureus* ATCC25923	*K. pneumoniae* ATCC29212	*P. aeruginosa* ATCC10145
4a	2.0 mg mL^−1^	(+) 12.00 ± 0.10	(+) 14.20 ± 0.05	(+) 10.0 ± 0.11	(+) 11.0 ± 0.09
	1.0 mg mL^−1^	(+) 4.50 ± 0.05	(+) 5.20 ± 0.05	(−)	(+) 4.50 ± 0.05
	0.5 mg mL^−1^	(−)	(−)	(−)	(−)
4c	2.0 mg mL^−1^	(+) 7.0 ± 0.10	(+) 4.5 ± 0.00	(+) 8.0 ± 0.40	(+) 8.5 ± 0.00
	1.0 mg mL^−1^	(−)	(−)	(−)	(−)
	0.5 mg mL^−1^	(−)	(−)	(−)	(−)
4f	2.0 mg mL^−1^	(+) 6.0 ± 0.0	(+) 3.50 ± 0.00	(+) 7.80 ± 0.60	(+) 9.80 ± 0.00
	1.0 mg mL^−1^	(−)	(−)	(−)	(+) 4.00 ± 0.00
	0.5 mg mL^−1^	(−)	(−)	(−)	(−)

a Values are given as mean ± standard error.

### Molecular docking

#### Docking and interaction studies with dihydropteroate synthase of *S. aureus*

**Fig. 4:**
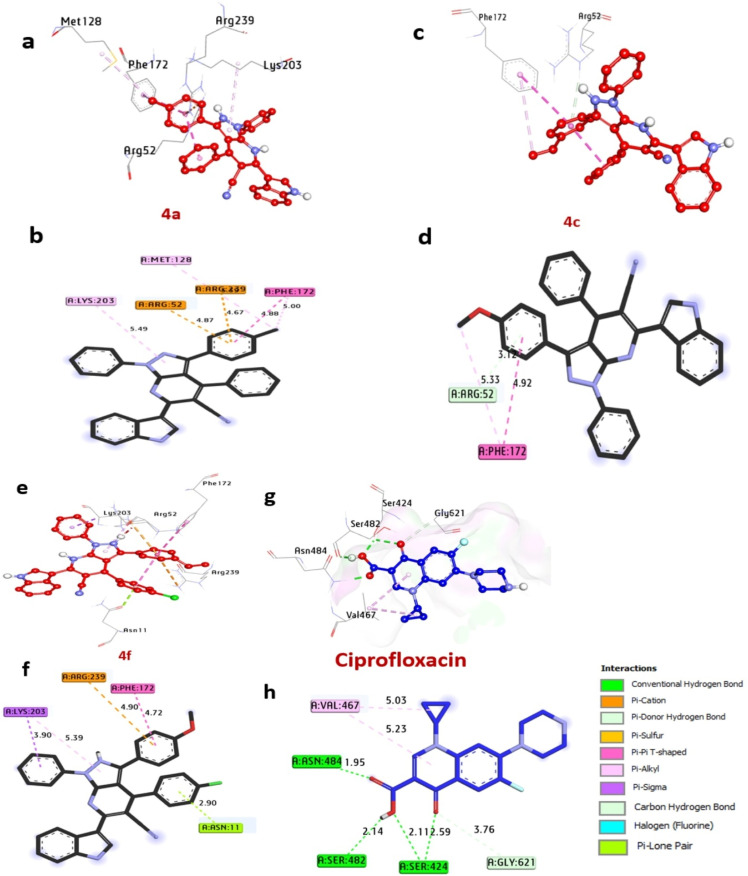
3D representations of the compound at the binding pocket of dihydropteroate synthase of *S. aureus* (PDB: ID 1AD4). (a and b) 4a, (c and d) 4c, (e and f) 4f, (g and h) ciprofloxacin.

Dihydropteroate synthase plays a significant is a key player in the production of folate, a necessary component for DNA synthesis and cellular metabolism. The synthesized compounds were evaluated for their binding energies can be viewed in Fig. 4. Compounds 4a, 4c, and 4f demonstrated favorable binding energies of −7.60, −7.40, and −7.60 kcal mol^−1^, respectively, outperforming ciprofloxacin (−6.40 kcal mol^−1^). No compounds was identified to establish hydrogen bonds. Additionally, they engaged in hydrophobic interactions within the enzyme's activity pocket, forming alkyl bonds with His241, Arg204, Lys203, Arg202, Met128, and Phe172, (C–H bond) with Arg239 and Arg52, (Pi-cation) with Arg239 and Arg52, (Pi–Pi stacked) with Phe172, (Pi-sigma) with Lys203, and (Pi-lone pair) with Asn11. Notably, amino acids Asn11, Val49, and Arg52 situated in the catalytic site were observed to enhance the binding affinity of these compounds. Consequently, compounds 4a, 4c, and 4f are anticipated to exhibit their antibacterial activity by effectively inhibiting the dihydropteroate synthase enzyme in *S. aureus* (Table 4). Our findings are similar to the study by Melk *et al.*,^51^ where computational analysis was conducted to elucidate the molecular interactions between promising compounds and enzymes as antimicrobial protein receptors.

**Table 4:** Molecular interactions of ligands with amino acids of dihydropteroate synthase of *S. aureus* (PDB: ID 1AD4)

Protein	Ligand	Hydrophilic interactions	Hydrophobic contacts			No. of H-bonds	No. of total bonds	Affinity kcal mol^−1^
		Residue (H-bond)	Length	Residue (bond type)	Length			
Dihydropteroate synthase of *S. aureus*	4a	—	—	Met128, (Pi-alkyl)	5.33	0	6	−7.60
				Phe172, (Pi-alkyl)	5.00			
				Lys203, (Pi-alkyl)	5.49			
				Arg239, (Pi-cation)	4.67			
				Arg52, (Pi-cation)	4.87			
				Phe172, (Pi–Pi stacked)	4.88			
	4c	—	—	Arg52, (C–H bond)	3.12	0	3	−7.40
				Phe172, (Pi-alkyl)	5.33			
				Phe172, (Pi–Pi stacked)	4.92			
	4f	—	—	Lys203, (Pi-alkyl)	5.39	0	5	−7.60
				Lys203, (Pi-sigma)	3.90			
				Arg239, (Pi-cation)	4.90			
				Phe172, (Pi–Pi stacked)	4.72			
				Asn11, (Pi-lone pair)	2.90			
	Ciprofloxacin	Val49, (H-bond)	2.74	Ser201, (Halogen)	3.02	2	8	−6.40
		Arg239 (H-bond)	2.18	Arg202, (Pi-alkyl)	5.11			
				His241, (Pi-cation)	4.69			
				Lys203, (Pi-alkyl)	5.37			
				Lys203, (CH-bond)	3.55			
				Arg219, (unfavorable)				

#### Docking and molecular interaction studies of LasR protein in *P. aeruginosa*

**Fig. 5:**
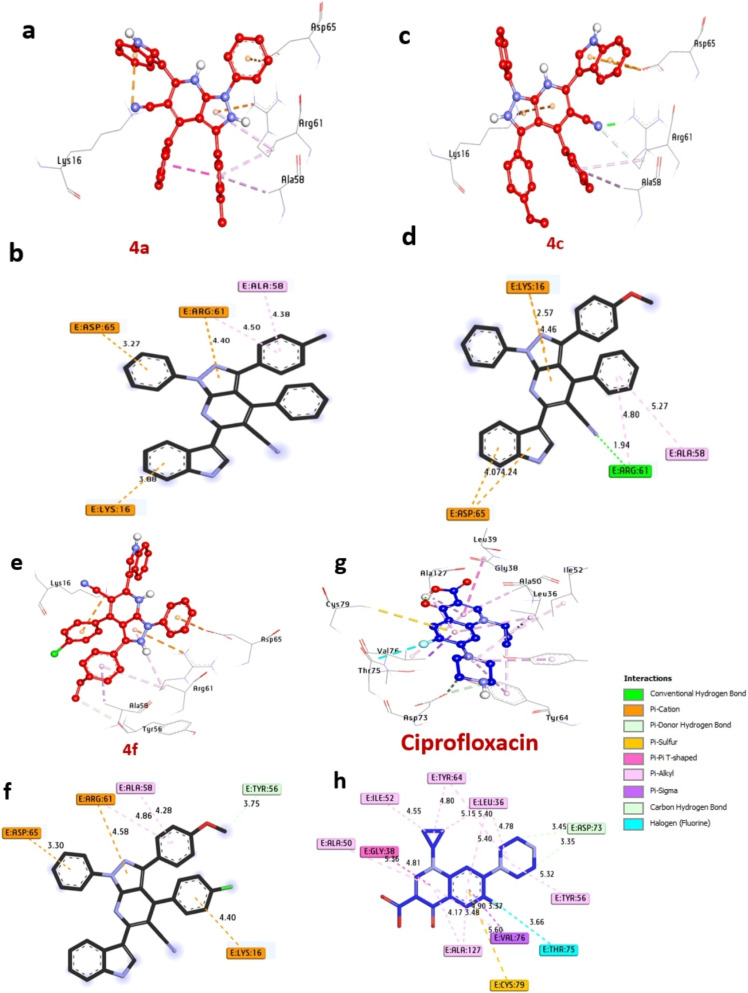
3D representations of compound at the binding pocket of LasR protein in *P. aeruginosa* (PDB: ID 2UV0): (a and b) 4a, (c and d) 4c, (e and f) 4f, (g and h) ciprofloxacin.

LasR protein in *P. aeruginosa* is a transcriptional regulator that plays a vital role in regulating the expression of virulence and pathogenicity. The docking results of molecules are summarized in Table 5 and visualized in Fig. 5. Notably, among the compounds under scrutiny, the most potent bacterial inhibitors, compounds 4a, 4c, and 4f, displayed significant affinity interactions with binding energies of −10.10, −11.60, and −10.90 kcal mol^−1^, respectively, surpassing ciprofloxacin (−8.50 kcal mol^−1^). These compounds were identified to establish hydrogen bonds with essential amino acids like Ser129 and Arg61, while also participating in diverse hydrophobic interactions within the protein's active site, including alkyl bonds with Arg61, and Ala58, (Pi-cation) with Asp65, Lys16, Arg61, (Pi-Anion) with Ala58, Asp65, Lys16, and Asp73, (C–H bond) with Tyr56. Furthermore, amino acids Ser129, Ala58, and Ser129 positioned within the catalytic site were observed to contribute to enhancing the binding affinity of these compounds (Table 5).The amalgamation of these results with the verified *in vitro* antibacterial activity outcomes suggests that compounds 4a, 4c, and 4f hold promise as potent bacterial inhibitors targeting the LasR protein in *P. aeruginosa*. Similar conclusions were drawn by Khidre *et al.*^43^ who demonstrated antimicrobial activity and provided *in silico* explanations using LasR protein in *P. aeruginosa* as a crucial enzyme and protein target for molecular docking.

**Table 5:** Molecular interactions of ligands with LasR protein in *P. aeruginosa*

Protein	Ligand	Hydrophilic interactions	Hydrophobic contacts			No. of H-bonds	No. of total bonds	Affinity kcal mol^−1^
		Residue (H-bond)	Length	Residue (bond type)	Length			
LasR protein in *P. aeruginosa*	4a	—	—	Asp65, (Pi-cation)	3.27	0	5	−10.10
				Lys16, (Pi-cation)	3.88			
				Arg61, (Pi-cation)	4.40			
				Arg61, (Pi-alkyl)	4.50			
				Ala58, (Pi-anion)	4.38			
	4c	Arg61, (H-bond)	1.94	Asp65, (Pi-anion)	4.07	1	7	−11.60
				Asp65, (Pi-anion)	4.24			
				Ala58, (Pi-alkyl)	5.27			
				Arg61, (Pi-alkyl)	4.80			
				Lys16, (Pi-anion)	2.57			
				Lys16, (Pi-anion)	4.46			
	4f	—	—	Tyr56, (C–H bond)	3.75	0	6	−10.90
				Arg61, (Pi-alkyl)	4.86			
				Ala58, (Pi-alkyl)	4.58			
				Arg61, (Pi-cation)	3.30			
				Asp65, (Pi-cation)	4.40			
				Lys16, (Pi-cation)	3.30			
	Ciprofloxacin	—	—	Asp73, (C–H bond)	3.45	0	15	−8.50
				Asp73, (C–H bond)	3.35			
				Tyr56, (Pi-alkyl)	5.32			
				Leu36, (Pi-alkyl)	4.78			
				Tyr64, (Pi-alkyl)	4.80			
				Ile52, (Pi-alkyl)	4.55			
				Ala50, (Pi-alkyl)	5.36			
				Ala127, (Pi-alkyl)	4.17			
				Thr75, (Halogen)	3.66			
				Val76, (Pi-sigma)	3.37			
				Gly38, (Pi–Pi T shaped)	4.81			
				Cys79, (Pi-sulfur)	5.60			

#### Docking and molecular interaction studies with DNA gyrase of *E.coli*

**Fig. 6:**
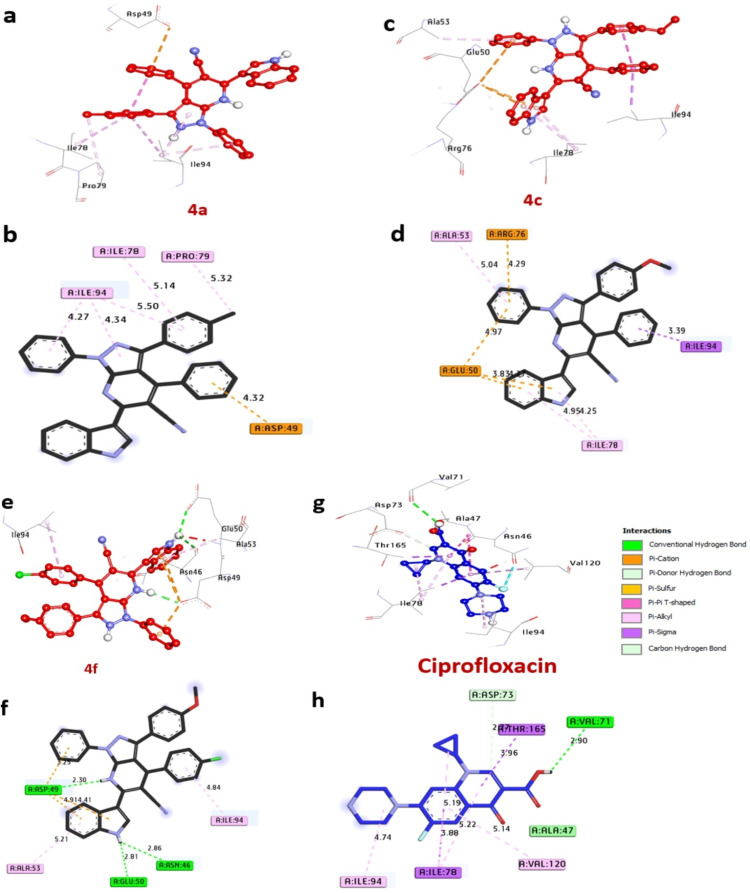
3D representations of compounds at the binding pocket of DNA gyrase of *E.coli* (PDB: ID 7P2M): (a and b) 4a, (c and d) 4c, (e and f) 4f, (g and h) ciprofloxacin.

The DNA gyrase, essential for DNA replication and transcription in *E. coli*. This examination, detailed in Table 6 and Fig. 6. These results spotlighted the most effective bacteria inhibitors: compounds 4a, 4c, and 4f, exhibiting remarkable affinity interactions of −9.0, −8.9, and −8.7 kcal mol^−1^, respectively, in contrast to ciprofloxacin (−7.20 kcal mol^−1^). Notably, only compound 4f demonstrated the formation of hydrogen bonds with crucial amino acids like Asp49, Asn46, and Glu50, while also engaging in diverse hydrophobic interactions within the enzyme's activity pocket, including alkyl bonds with Ile78, Pro79, Ile94, Ala53, and Val120, (Pi-cation) with Arg76, Asp49, and (Pi-sigma) with Ile94. Moreover, amino acids Asp49, Glu50, and Asn46 located at the catalytic site were observed to contribute to enhancing the binding affinity of these compounds. The comprehensive results from the docking analysis suggest that compounds 4a, 4c, and 4f have the potential to act as potent inhibitors of bacterial growth by targeting the DNA gyrase enzyme. These results were similar to Sroor *et al.*,^44^ who used molecular docking and in silicon screening of compounds against DNA gyrase as antimicrobial protein receptor.

**Table 6:** Molecular interactions of ligands with amino acids of DNA gyrase of *E.coli* (PDB: ID 7P2M)

Protein	Ligand	Hydrophilic interactions		Hydrophobic contacts		No. of H-bonds	No. of total bonds	Affinity kcal mol^−1^
		Residue (H-bond)	Length	Residue (bond type)	Length			
DNA gyrase of *E.coli* (PDB: ID 7P2M)	4a	—	—	Pro79, (alkyl)	5.32	0	6	−9.0
				Ile78, (alkyl)	5.14			
				Ile94, (alkyl)	5.50			
				Ile94, (alkyl)	4.34			
				Ile94, (alkyl)	4.27			
				Asp49, (pi-cation)	4.32			
	4c	—	—	Ala53, (alkyl)	5.04	0	8	−8.9
				Ile78, (alkyl)	4.95			
				Ile78, (alkyl)	4.25			
				Ile94, (pi-sigma)	3.39			
				Arg76, (pi-cation)	4.29			
				Glu50, (pi-cation)	4.97			
				Glu50, (pi-cation)	3.83			
	4f	Asp49, (H-bond)	2.30	Ile94, (alkyl)	4.84	3	8	−8.7
		Asn46, (H-bond)	2.86	Ala53, (alkyl)	5.21			
		Glu50, (H-bond)	2.81	Asp49, (pi-cation)	2.30			
				Asp49, (pi-cation)	4.91			
				Asp49, (pi-cation)	4.41			
	Ciprofloxacin	Glu50, (H-bond)	2.90	Ile94, (alkyl)	4.74	1	8	−7.20
				Ile78, (alkyl)	5.22			
				Ile78, (alkyl)	5.19			
				Val120, (alkyl)	5.14			
				Ile78, (pi-sigma)	3.88			
				Thr165, (pi-sigma)	3.96			
				Asp73, (C–H bond)	2.77			

#### Docking and interaction studies of KPC-2 carbapenemase of *K. pneumoniae*

**Fig. 7:**
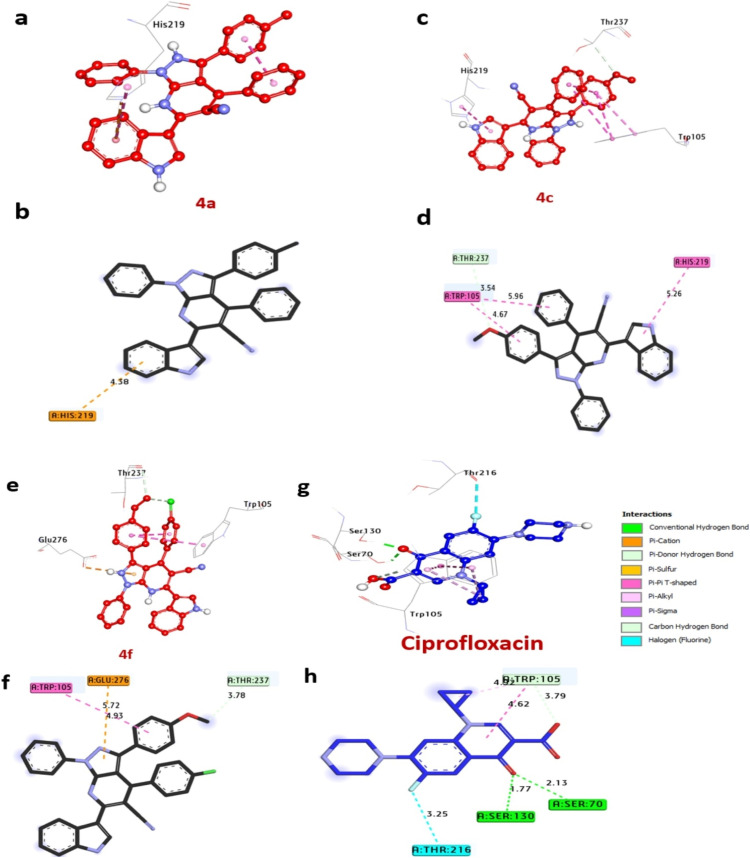
3D representations of compounds at the binding pocket of KPC-2 carbapenemase of *K. pneumoniae* (PDB: ID 2OV5): (a and b) 4a, (c and d) 4c, (e and f) 4f, (g and h) ciprofloxacin.

Molecular docking studies were conducted to elucidate the potential binding modes and affinities of the synthesized compounds against the KPC-2 carbapenemase, a key enzyme conferring antibiotic resistance in *K. pneumoniae*. The docking outcomes involving molecules and the ciprofloxacin are extensively outlined in Table 7 and Fig. 7. Notably, among the compounds examined, 4a, 4c, and 4f demonstrated significant affinity interactions of −7.0, −7.4, and −7.7 kcal mol^−1^, respectively, in contrast to the ciprofloxacin (−7.3 kcal mol^−1^). Notably, compound 4f emerged as the most promising candidate from our series, exhibiting a superior predicted binding affinity of −7.7 kcal mol^−1^. Instead of forming traditional hydrogen bonds, 4f stabilized its position exclusively through hydrophobic and Pi-based interactions, including a Pi-cation interaction with Gln226, a Pi–Pi T-shaped interaction with Thr105, and a C–H bond with Thr232. This suggests that 4f fits snugly into the hydrophobic sub-pockets of the binding site, with the energy from this extensive van der Waals and electrostatic contacts compensating for the lack of hydrogen bonds. Similarly, compound 4c also showed a favorable binding affinity of −7.4 kcal mol^−1^. Its binding was characterized by four hydrophobic interactions, dominated by a cluster of Pi–Pi T-shaped interactions with Thr102, Thr103, and Thr105, alongside a C–H bond with Thr232. In contrast, compound 4a was the weakest binder in the series (affinity = −7.0 kcal mol^−1^), forming only a single Pi-cation interaction, which was insufficient for strong stabilization. In conclusion, the docking simulations successfully identified 4f and 4c as high-affinity ligands for KPC-2 carbapenemase. Their potent, hydrophobically-driven binding modes present a novel approach to enzyme inhibition, differing significantly from the mixed polar/non-polar strategy of ciprofloxacin. The superior theoretical affinity of 4f makes it a compelling lead compound for further investigation and development as a potential KPC-2 inhibitor. These interactions were observed to enhance the binding affinity of these compounds, particularly with amino acids Thr216, Thr237, and Trp105 situated at the catalytic site. The collective findings from the docking investigations, along with the validated *in vitro* antibacterial activity results, indicate the potential of compounds 4a, 4c, and 4f as promising inhibitors of *K. pneumoniae*. Similar conclusions were drawn by Jawhari *et al.*,^42^ who demonstrated antimicrobial activity and provided *in silico* explanations using KPC-2 carbapenemase within *K. pneumonia* as a critical enzyme and protein target for molecular docking.

**Table 7:** Molecular interactions of ligands with amino acids of KPC-2 carbapenemase of *K. pneumoniae* (PDB: ID 2OV5)

Protein	Ligand	Hydrophilic interactions		Hydrophobic contacts		No. of H-bonds	No. of total bonds	Affinity kcal mol^−1^
		Residue (H-bond)	Length	Residue (bond type)	Length			
KPC-2 carbapenemase of *K. pneumoniae*	4a	—	—	His219, (Pi-cation)	4.38	0	1	−7.0
	4c	—	—	Trp105, (Pi–Pi T shaped)	5.96	0	4	−7.4
				Trp105, (Pi–Pi T shaped)	4.67			
				His219, (Pi–Pi T shaped)	5.26			
				Thr237, (C–H bond)	3.54			
	4f	—	—	Trp105, (Pi–Pi T shaped)	4.93	0	3	−7.7
				Glu276, (Pi-cation)	5.72			
				Thr237, (C–H bond)	3.78			
	Ciprofloxacin	Ser70, (H-bond)	2.13	Thr216, (Halogen)	3.25	2	6	−7.3
				Trp105, (Pi-alkyl)	4.32			
		Ser130, (H-bond)	1.77	Trp105, (Pi–Pi T shaped)	4.62			
				Trp105, (C–H bond)	3.79			

#### *In silico* ADMET prediction of synthesized compounds

**Fig. 8:**
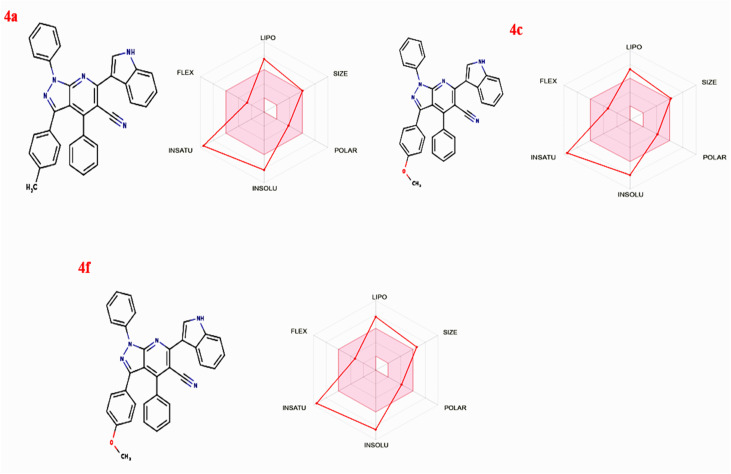
Oral bio-availability graph for compounds 4a, 4c, and 4f produced with the help of the Swiss ADME tool.

**Fig. 9:**
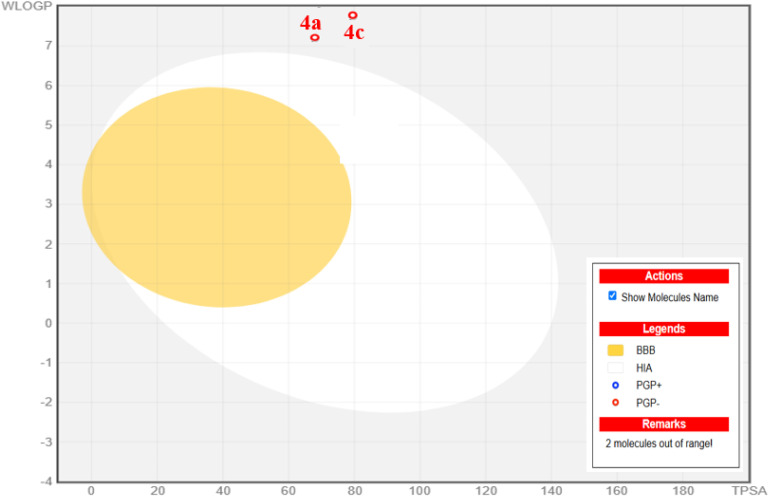
The boiled egg model for 4a, 4c, and 4f.

The physiochemical and ADMET evaluations presented in Table 8 and Fig. 8 highlight the promising characteristics of the compounds in our study. These compounds, with molecular weights around 500, align with Lipinski's rules, indicating their small size, ease of transfer, and efficient absorption. They possess a favorable number of rotatable bonds (4–5), which is essential for structural flexibility, and maintain a balanced ratio of hydrogen bond acceptors (HBA) and donors (HBD) (<5 HBA, <3 HBD), enhancing their potential for oral bioavailability. Regarding lipophilicity and water solubility, most compounds show limited water solubility, which is highly soluble. The lipophilicity parameter XLOGP3 ranges from 4.13 to 7.89, which is acceptable. Although all compounds exhibit high theoretical bioavailability, they face challenges in crossing the blood–brain barrier and show low intestinal absorption. Comprehensive drug-likeness assessments using various criteria, including Lipinski, Ghose, Veber, Muegge, and Egan rules reveal that some compound meets the stringent requirements, highlighting its favorable physicochemical profile for potential drug development. Despite indications of tumorigenicity, mutagenicity, irritancy, or reproductive toxicity in most compounds, their topological polar surface area (TPSA) values (70.29–79.52) suggest favorable absorption in the gastrointestinal tract and oral bioavailability. Notably, compound 4c stands out with higher drug scores compared to others, indicating its promising potential as a drug-like agent with antibacterial properties (Table 9). In summary, these findings collectively suggest that the compounds hold significant promise as potential drug candidates with high theoretical bioavailability, particularly in the field of antibacterial therapeutics Fig. 9.

**Table 8:** Prediction of pharmacokinetics and physicochemical properties of compounds

Characteristics	ID	4a	4c	4f
Physicochemical properties	MW	501.58	517.58	552.02
	Atoms	39	40	41
	Heavy atoms	36	36	36
	Csp^3^	0.03	0.03	0.03
	Rotatable bonds	4	5	5
	H-BA	3	4	4
	H-BD	1	1	1
	Molar refractivity	156.71	158.24	163.25
	TPSA	70.29	79.52	79.52
Lipophilicity and water solubility	iLOGP	4.13	4.07	4.31
	XLOGP3	7.65	7.26	7.89
	WLOGP	8.08	7.78	8.44
	MLOGP	5.03	4.21	4.66
	Silicos-IT log *P*	7.55	7.09	7.72
	*C* log *P*	6.49	6.08	6.6
	ESOL log *S*	−8.19	−7.96	−8.55
	Ali log *S*	−8.97	−8.75	−9.41
	Silicos-IT class	Poorly	Poorly	Poorly
Pharmacokinetics	GI absorption	Low	Low	Low
	BBB permeant	No	No	No
	Pgp substrate	No	No	No
	CYP1A2 inhibitor	No	No	No
	CYP2C19 inhibitor	No	No	No
	CYP2C9 inhibitor	No	No	No
	CYP2D6 inhibitor	No	No	No
	CYP3A4 inhibitor	No	No	No
	Skin permeation	−3.93	−4.3	−4.07
Drug likeness	Lipinski	2	2	2
	Ghose	3	3	3
	Veber	0	0	0
	Egan	1	1	1
	Muegge	1	1	1
	Bioavailability	0.17	0.17	0.17
	Lead likeness	2	2	2

**Table 9:** Prediction of toxicity risks and physicochemical properties of compounds 4a, 4c, and 4f

Ligand	Toxicity risks				Physicochemical properties					
	Mutagenic	Tumorigenic	Irritant	Reproductive	*C* log *P*	Solubility	Molecular weight	TPSA	Drug likeness	Drug score
4a	(−)	(−)	(−)	(−)	6.81	−10.19	501.0	70.29	−1.40	0.13
4c	(−)	(−)	(−)	(−)	6.39	−9.86	517.0	79.52	0.07	0.17
4f	(−)	(−)	(−)	(−)	7.0	−10.60	551.0	79.52	0.78	0.16

## Experimental

### Chemistry

#### Materials and instrumentation

All chemicals purchased from Sigma-Aldrich were used as obtained without further purification. TLC (thin-layer chromatography) was used to monitor chemical reactions using eluent (petroleum ether : ethyl acetate, 8 : 2). For TLC, Silica gel 60 F 254 was applied to aluminum sheets (20 × 20 cm). Purification of the products was performed under flash conditions using a column chromatography (silica gel 60, 0.040–0.063 mm). Bruker VERTEX 80v FTIR Spectrometer was used to measure FTIR spectra (*ν*_max_ in cm^−1^, KBr). ^1^H and ^13^C NMR spectra were recorded on a Bruker High-Performance Digital FT NMR spectrometer, Avance III (400 MHz for ^1^H and 100 MHz for ^13^C NMR). ^1^H and ^13^C NMR signals were referenced to tetramethylsilane (TMS) and the solvent shifts of DMSO-d_6_. The abbreviations for reporting ^1^H NMR data were denoted as follows: s, singlet; br s, broad singlet; d, doublet; t, triplet; m, multiplet. Values of the coupling constant (*J*) were recorded in hertz (Hz). Electron impact mass spectra were measured using a DI Analysis Shimadzu QP-2010 plus (70 eV). An Elemental Analyzer CHNS-932 (LECO) was used for elemental analyses.

##### General procedure for preparation of 6-(1*H*-indol-3-yl)-1,4,3-substituted-1*H*-pyrazolo[3,4-*b*]pyridine-5-carbonitrile 4a–f^13^

A mixture of an equimolar amount of 3-indolyl-3-oxopropanenitrile (1), aromatic aldehyde 2 and 1*H*-pyrazol-5-amine 3 in absolute ethanol (20 mL) in the presence of triethylamine (TEA) as a catalyst was heated at 80 °C for 5–12 h (the reaction progress was monitored by TLC). Upon completion, the reaction mixture was cooled to room temperature. The formed product was filtered, dried and purified using a column chromatography (silica gel 60, 0.040–0.063 mm) to afford compound 4a–f.

##### 6-(1*H*-Indol-3-yl)-1,4-diphenyl-3-(*p*-tolyl)-1*H*-pyrazolo[3,4-*b*]pyridine-5-carbonitrile (4a)

Yellow powder; (yield 75%); m.p. 290–291 °C; IR (KBr, *ν*_max_/cm^−1^):3377 (NH), 3047 (aromatic H), 2226 (C≡N); ^1^H NMR (DMSO-d_6_) *δ* ppm: 2.23 (s, 3H, CH_3_), 7.27–7.76 (m, 17H, Ar–H), 8.32 (d, 1H, Ar–H), 8.47 (s, 1H, pyrrole-H), 11.64 (s, 1H, NH, D_2_O exchangeable); ^13^C NMR (DMSO-d_6_) *δ* ppm: 21.2 (CH_3_), 111.2–131.8 (C_Ar_), 147.4 (C–N), 151.2 (C–N), 152.5 (C–N), 157.3 (C–N); MS, *m*/*z* (%): 501 (M^+^, 81); anal. calcd. For C_34_H_23_N_5_ (501.59) (%): C, 81.42; H, 4.62; N, 13.96. Found (%): C, 81.50; H, 4.59; N, 13.91.

##### 3-(4-Chlorophenyl)-6-(1*H*-indol-3-yl)-1,4-diphenyl-1*H*-pyrazolo[3,4-*b*]pyridine-5-carbonitrile (4b)

Yellow powder; (yield 77%); m.p. 280–281 °C; IR (KBr, *ν*_max_/cm^−1^): 3380 (NH), 3045 (aromatic H), 2222 (C≡N); ^1^H NMR (DMSO-d_6_) *δ* ppm: 7.26–7.78 (m, 17H, Ar–H), 8.33 (d, 1H, Ar–H), 8.46 (s, 1H, pyrrole-H), 11.75 (s, 1H, NH, D_2_O exchangeable); ^13^C NMR (DMSO-d_6_) *δ* ppm: 110.8–134.2 (C_Ar_), 146.1 (C–N), 149.9 (C–N), 151.4 (C–N), 155.1 (C–N); MS, *m*/*z* (%): 521 (M^+^, 20); anal. calcd. For C_33_H_20_ClN_5_ (522.01) (%): C, 75.93; H, 3.86; Cl, 6.79; N, 13.42. Found (%): C, 76.00; H, 3.84; Cl, 6.76; N, 13.40.

##### 6-(1*H*-Indol-3-yl)-3-(4-methoxyphenyl)-1,4-diphenyl-1*H*-pyrazolo[3,4-*b*]pyridine-5-carbonitrile (4c)

Brown powder; (yield 76%); m.p. 285–286 °C; IR (KBr, *ν*_max_/cm^−1^): 3371 (NH), 3050 (aromatic H), 2217 (C≡N); ^1^H NMR (DMSO-d_6_) *δ* ppm: 3.68 (s, 3H, OCH_3_), 6.58–7.61 (m, 17H, Ar–H), 8.31 (m, 1H, Ar–H), 8.46 (s, 1H, pyrrole-H), 11.89 (s, 1H, NH, D_2_O exchangeable); ^13^C NMR (DMSO-d_6_) *δ* ppm: 65.9 (OCH_3_), 110.6–134.9 (C_Ar_), 148.0 (C–N), 151.6 (C–N), 153.4 (C–N), 166.4 (C–O); MS, *m*/*z* (%): 517 (M^+^, 68); anal. calcd. For C_34_H_23_ClN_5_O (517.59) (%): C, 78.90; H, 4.48; N, 13.53. Found (%): C, 78.83; H, 4.52; N, 13.59.

##### 4-(4-Chlorophenyl)-6-(1*H*-indol-3-yl)-1-phenyl-3-(*p*-tolyl)-1*H*-pyrazolo[3,4-*b*]pyridine-5-carbonitrile (4d)

Yellow powder; (yield 77%); m.p. 286–287 °C; IR (KBr, *ν*_max_/cm^−1^): 3370 (NH), 3055 (aromatic H), 2224 (C≡N); ^1^H NMR (DMSO-d_6_) *δ* ppm: 2.25 (s, 3H, CH_3_), 6.92–7.63 (m, 16H, Ar–H), 8.32 (d, 1H, Ar–H), 8.46 (s, 1H, pyrrole-H), 11.91 (s, 1H, NH, D_2_O exchangeable); ^13^C NMR (DMSO-d_6_) *δ* ppm: 21.3 (CH_3_), 110.9–131.7 (C_Ar_), 147.3 (C–N), 150.8 (C–N), 152.1 (C–N), 156.1 (C–N); MS, *m*/*z* (%): 535 (M^+^, 88); anal. calcd. For C_34_H_22_ClN_5_ (536.16) (%): C, 76.18; H, 4.14; Cl, 6.61; N, 13.07. Found (%): C, 76.23; H, 4.09; Cl, 6.66; N, 13.02.

##### 3,4-Bis(4-chlorophenyl)-6-(1*H*-indol-3-yl)-1-phenyl-1*H*-pyrazolo[3,4-*b*]pyridine-5-carbonitrile (4e)

Yellow powder; (yield 75%); m.p. 292–293 °C; IR (KBr, *ν*_max_/cm^−1^): 3380 (NH), 3066 (aromatic H), 2219 (C≡N); ^1^H NMR (DMSO-d_6_) *δ* ppm: 7.10–7.32 (m, 16H, Ar–H), 8.29 (d, 1H, Ar–H), 8.46 (s, 1H, pyrrole-H), 11.94 (s, 1H, NH, D_2_O exchangeable); ^13^C NMR (DMSO-d_6_) *δ* ppm: 111.0–137.0 (C_Ar_), 150.8 (C–N), 151.6 (C–N), 156.3 (C–N); MS, *m*/*z* (%): 555 (M^+^, 73); anal. calcd. For C_33_H_19_Cl_2_N_5_ (556.45) (%): C, 71.23; H, 3.44; Cl, 12.74; N, 12.59. Found (%):C, 71.31; H, 3.46; Cl, 12.69; N, 12.54.

##### 4-(4-Chlorophenyl)-6-(1*H*-indol-3-yl)-3-(4-methoxyphenyl)-1-phenyl-1*H*-pyrazolo[3,4-*b*]pyridine-5-carbonitrile (4f)

Brown powder; (yield 75%); m.p. 295–296 °C; IR (KBr, *ν*_max_/cm^−1^): 3379 (NH), 3082 (aromatic H), 2219 (C≡N); ^1^H NMR (DMSO-d_6_) *δ* ppm: 3.70 (s, 3H, OCH_3_), 6.90–7.65 (m, 16H, Ar–H), 8.31 (d, 1H, Ar–H), 8.45 (s, 1H, pyrrole-H), 11.85 (s, 1H, NH, D_2_O exchangeable); ^13^C NMR (DMSO-d_6_) *δ* ppm: 69.3 (OCH_3_), 110.3–134.7 (C_Ar_), 147.3 (C–N), 150.8 (C–N), 152.1 (C–N), 166.1 (C–O); MS, *m*/*z* (%): 551 (M^+^, 85); anal. calcd. For C_34_H_22_ClN_5_O (552.03) (%): C, 73.98; H, 4.02; Cl, 6.42; N, 12.69. Found (%): C, 74.06; H, 3.95; Cl, 6.46; N, 12.64.

## Evaluation of antioxidant activity of compounds

According to Mansoor *et al.*,^52^ DPPH was used to study the free radical-scavenging activities of compounds one mL of the compounds was added to 1.0 mL methanolic solution of 0.3 mM DPPH. The mixture was shaken and left in a dark box for 30 minutes at room temperature (30 °C). The absorbance of the resulting solution was measured at 517 nm. The inhibitory percentage of DPPH was calculated according to the following equation:
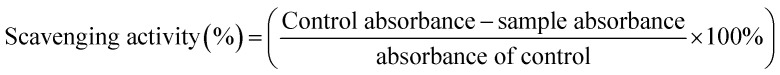


The half-maximal inhibitory concentration (IC_50_) for antioxidant activity was calculated from the dose–response data of the DPPH assay and performed using GraphPad Prism software, version 9.0.

## Evaluation of antibacterial activity of compounds

All compounds were screened against pathogenic bacterial strains (*Escherichia coli*, *Staphylococcus aureus*, *K. pneumoniae*, and *Pseudomonas aeruginosa*) using the well diffusion method described by Abdelrazik *et al.*,^53^ The bacterial strains were obtained from the Microbial Genetics Lab at the National Research Centre in Egypt. The microbes were sub-cultured in nutrient broth and incubated for 24 hours at 37 °C and 120 rpm. Each strain was then swabbed onto Mueller–Hinton agar using sterile cotton swabs. Subsequently, 50 µl of each compound (at a concentration of 2 mM in DMSO) of each compound was inoculated into the wells. After 24 hours of incubation at 37 °C, the zones of inhibition (ZOI) were measured in millimeters (mm) using a zone scale. Ciprofloxacin was used as a standard positive control antibiotic for all bacterial strains.

## Computational methods

### Molecular docking

All protein receptors were sourced from the RCSB database as detailed in Table 10. The target protein structures were then preprocessed using PyMOL, which involved removing water molecules, ions, and existing ligands. The structures of the compounds were drawn using BIOVIA Draw. Open Babel O'Boyle *et al.*,^54^ was used to convert each compound into the mol2 format. Following this, AutoDock tools were employed to convert the molecules into the pdbqt format. Before docking, ligand-centered maps were generated using AutoDock Vina Eberhardt *et al.*,^55^ The Discovery Studio program was used to analyze the 2-D interactions between the target proteins and the ligands. The Swiss ADME server was used to calculate the compound's physicochemical characteristics and absorption, distribution, metabolism, excretion, and toxicity.

**Table 10:** Molecular docking targets of bacterial strains, PDB ID's, active site coordinates, native co-crystalized ligands, and reference ligands

Organism		Protein targets	PDB ID	Active site coordinates			Reference ligands
				X	Y	Z	
*K. pneumoniae*	G +ve	KPC-2 carbapenemase	2OV5	40.3	1.52	8.17	Ciprofloxacin
*E.coli*	G−ve	DNA gyrase	7P2M	4.36	33.47	−14.16	Ciprofloxacin
*S. aureus*	G+ve	Dihydropteroate synthase of *S.aureus*	1AD4	33.4	5.95	37.9	Ciprofloxacin
*P. aeruginosa*	G−ve	LasR an activator of exotoxin A expression in *P. aeruginosa*	2UV0	24.37	13.79	81.52	Ciprofloxacin

## Conclusions

A series of 1-phenyl-1*H*-pyrazolo[3,4-*b*]pyridine-5-carbonitriles 4a–f were prepared through one-pot three-component reaction. The new derivatives were analyzed using spectroscopic techniques and elemental analysis. The newly synthesized compounds were subsequently evaluated for their antimicrobial and antioxidant activities, with molecular docking used to investigate their interactions with key proteins. Compounds 4a, 4c, and 4f exhibited strong antimicrobial effects against various bacterial strains, including *E. coli*, *S. aureus*, *P. aeruginosa*, and *K. pneumoniae*. Their antioxidant properties were also assessed using DPPH radical scavenging methods. Molecular docking screenings revealed favorable binding energies, indicating the compounds' potential to effectively inhibit a vital enzyme. Additionally, *in silico* ADMET and drug-likeness screenings showed compounds comply with Lipinski's rules, underscoring their potential as oral bioavailable drug candidates with favorable properties.

## Author contributions

HSK: investigation, supervision, methodology, and writing original draft. AFE: investigation, methodology, and writing original draft AHS: investigation, methodology, writing, review and editing.

## Conflicts of interest

The authors declare no conflict of interest.

## Supplementary Material

RA-015-D5RA07372C-s001

## Data Availability

The data that support the findings of this study are available in the supplementary information (SI) of this article. Supplementary information is available. See DOI: https://doi.org/10.1039/d5ra07372c. Supplementary data has been deposited with Protein Data Bank.^56*a*–*d*^ Enzymes and corresponding PDB ID, including KPC-2 carbapenemas of *K. pneumoniae* with (PDB.ID: 2OV5), DNA Gyrase of *E.coli* with (PDB.ID: 7P2M), Dihydropteroate synthase of *S. aureus* with (PDB.ID: 1AD4), and LasR an activator of exotoxin A expression in *P. aeruginosa* with (PDB.ID: 2UV0) were used for the docking study.
