# Supplementary material for: Enhanced antibacterial and antioxidant capabilities using indole-modified 1-phenyl-1H-pyrazolo[3,4-b]pyridine-5-carbonitrile derivatives, molecular docking evaluation and in silico ADMET prediction
Source: RSC Adv. 2025 Dec 1;15(55):47255–70. doi: 10.1039/d5ra07372c (PMC12668186; doi:10.1039/d5ra07372c)
Supplement: RA-015-D5RA07372C-s001 [file RA-015-D5RA07372C-s001.pdf]

This journal is © The Royal Society of Chemistry 2025

SUPPLEMENTARY INFORMATION

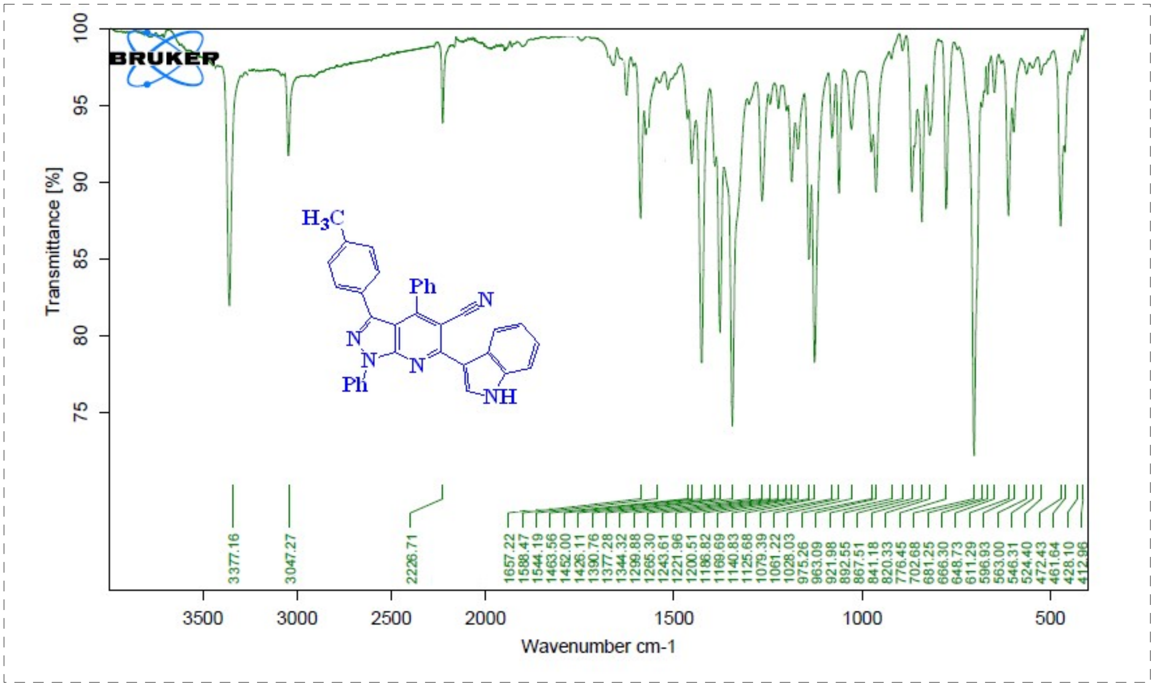

Figure S1. IR spectrum of compound **4a**

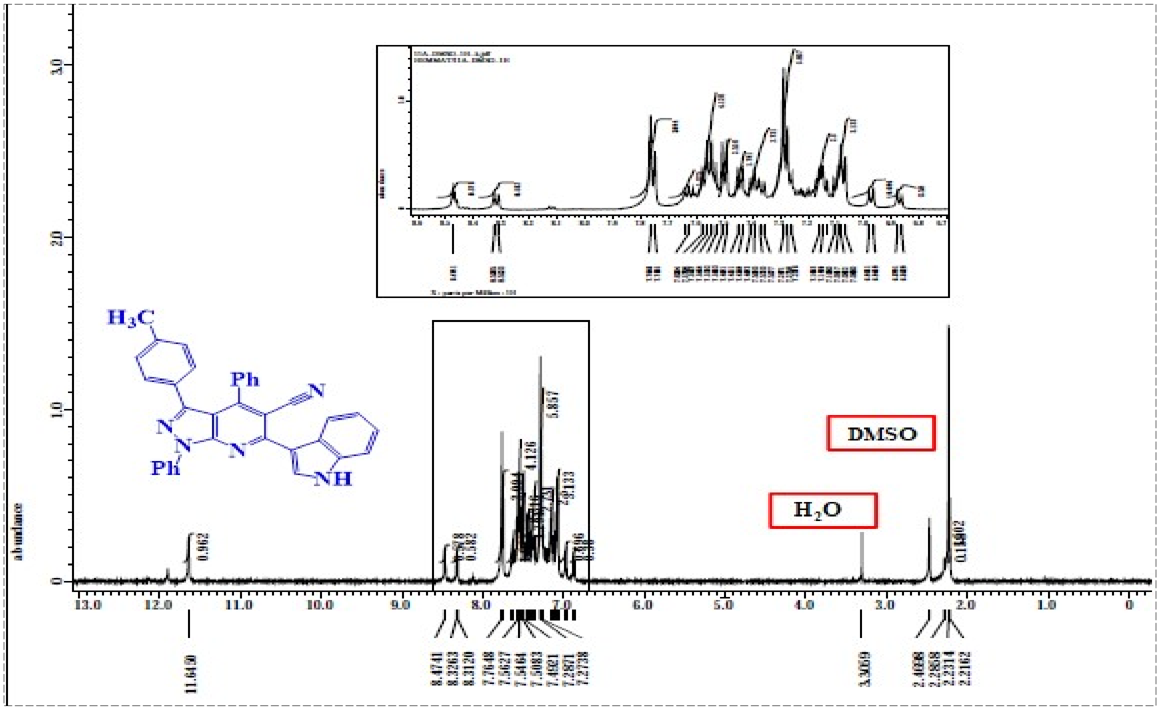

Figure S2. ^{1}H NMR spectrum of compound **4a**

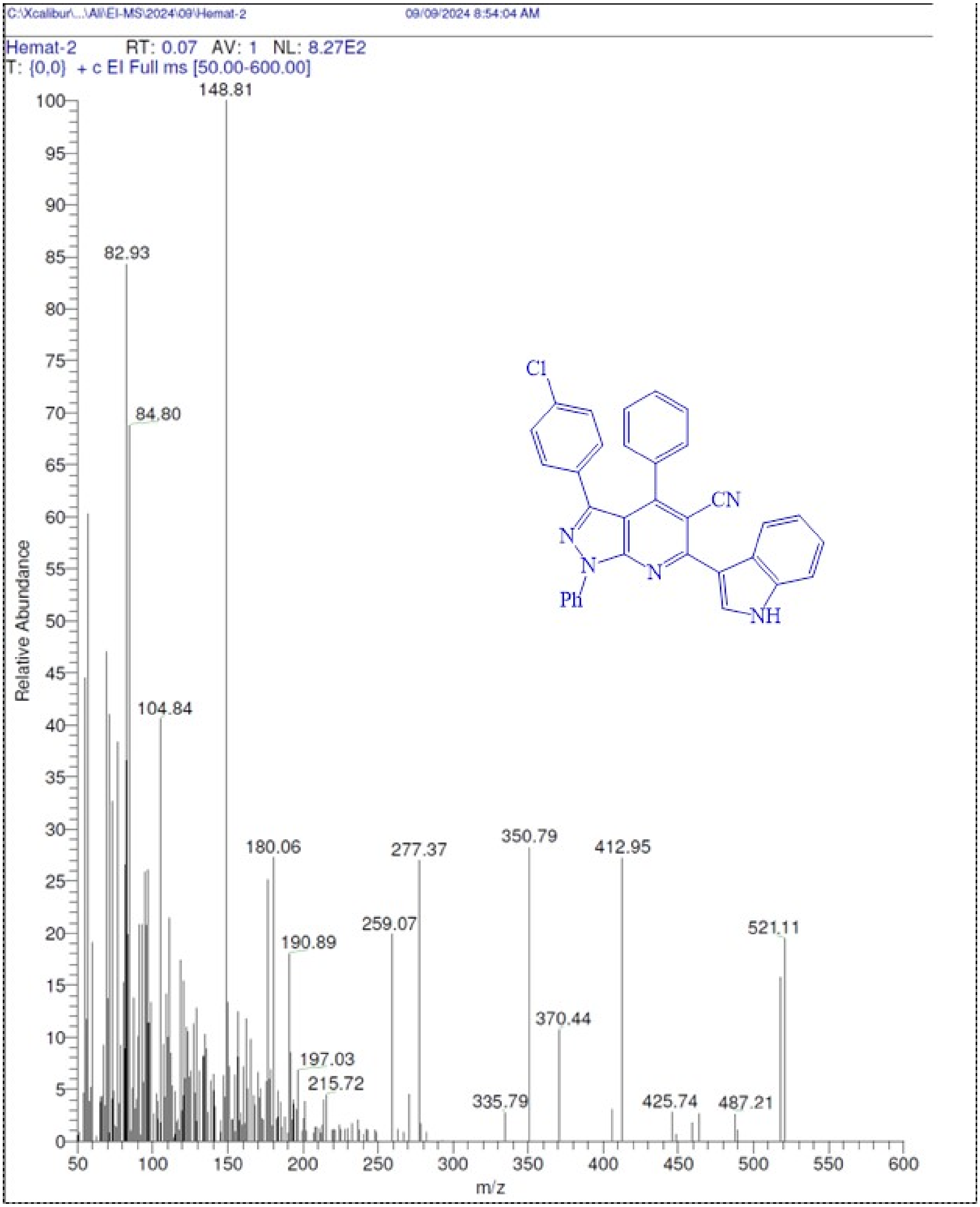

Figure S3. Mass spectrum of compound **4b**

MS, m/z (%): 521 (M^+, 20); Anal. Calcd. For $C_{33}H_{20}ClN_5$ (522.01)

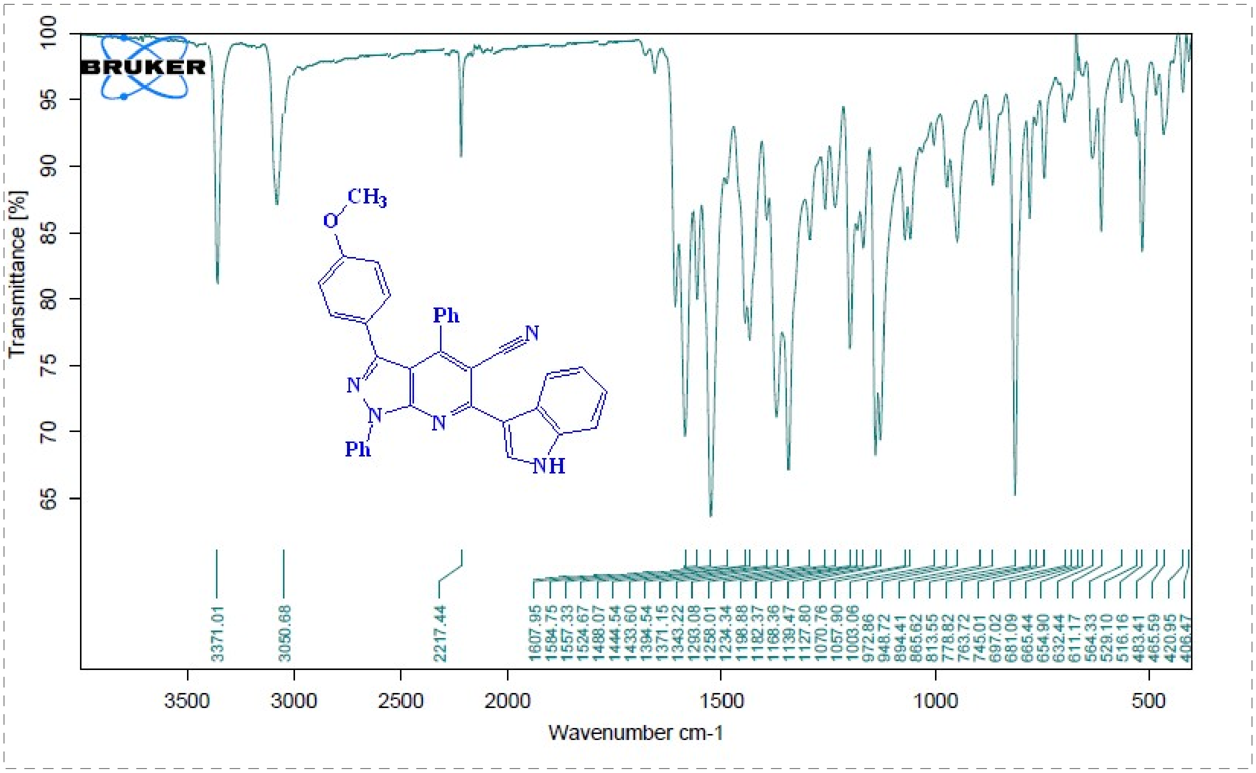

Figure S4. IR spectrum of compound **4c**

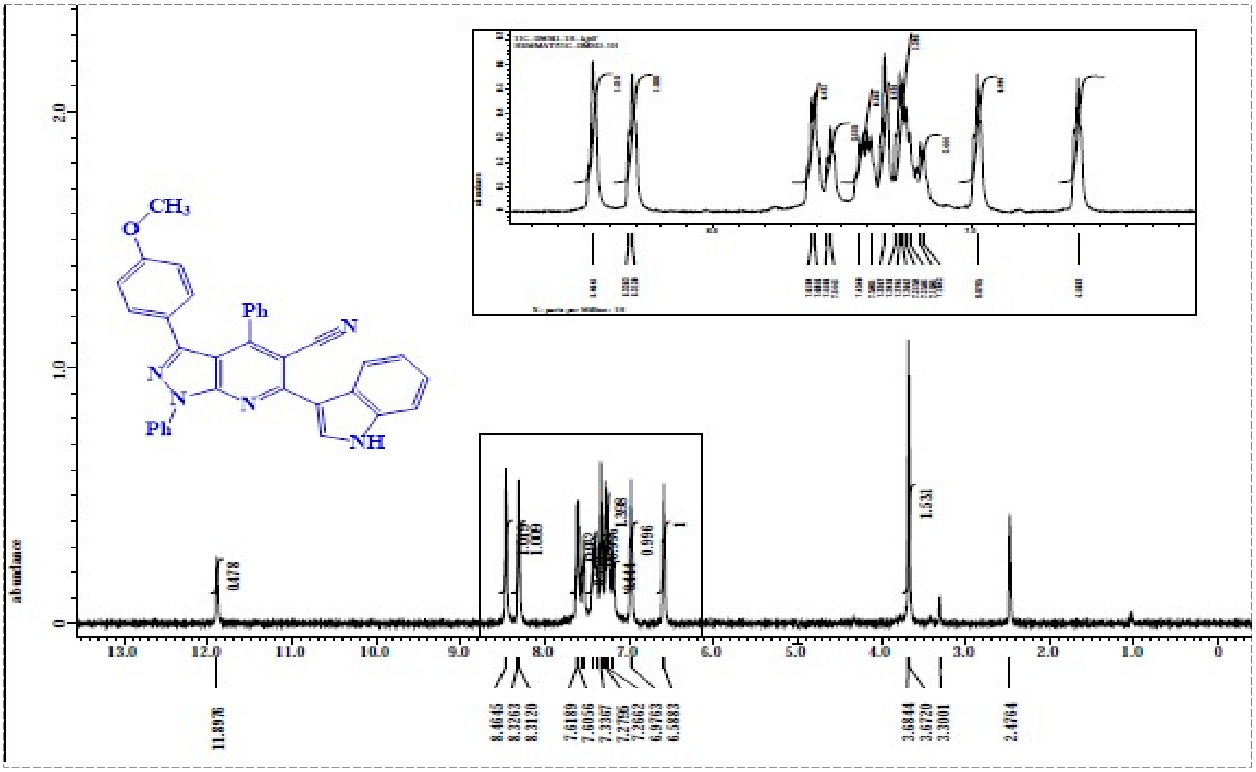

Figure S5. ^{1}H NMR spectrum of compound **4c**

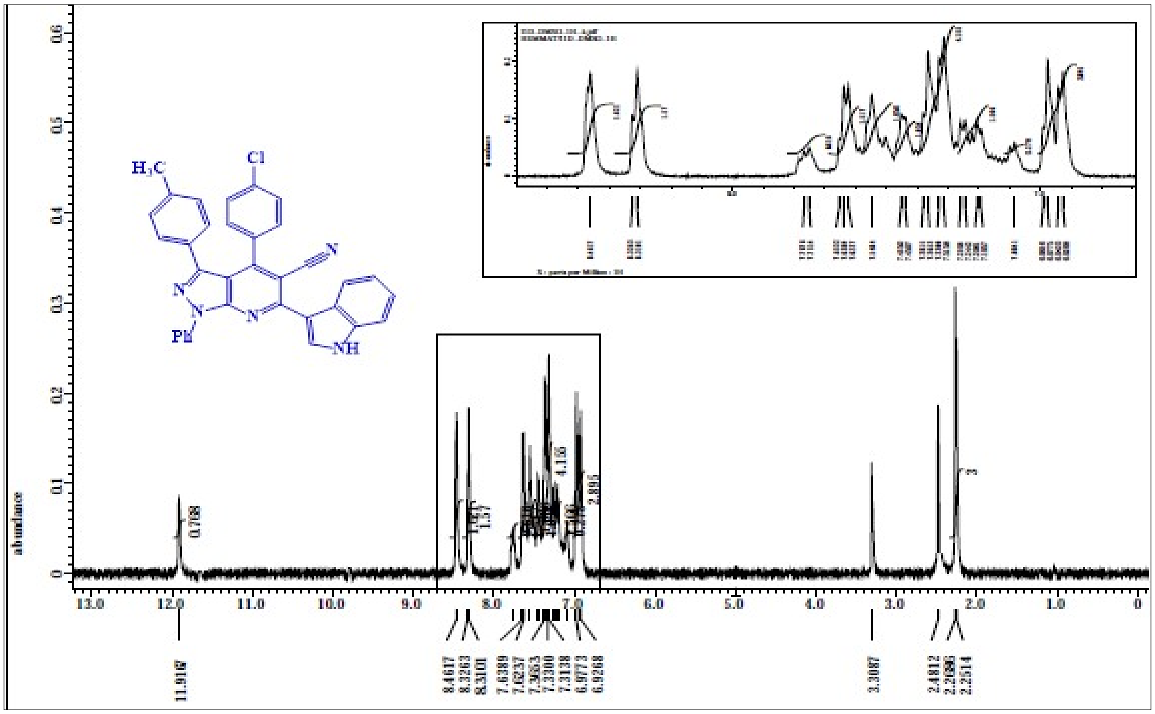

Figure S6. ^{1}H NMR spectrum of compound **4d**

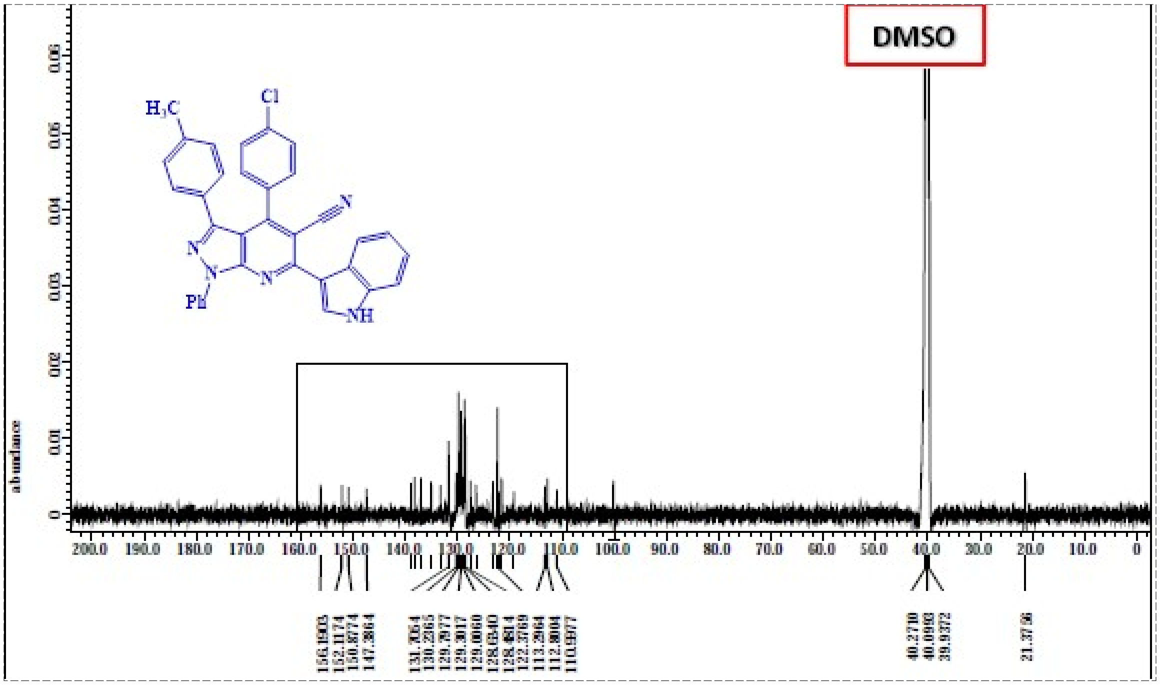

Figure S7. ^{13}C NMR spectrum of compound **4d**

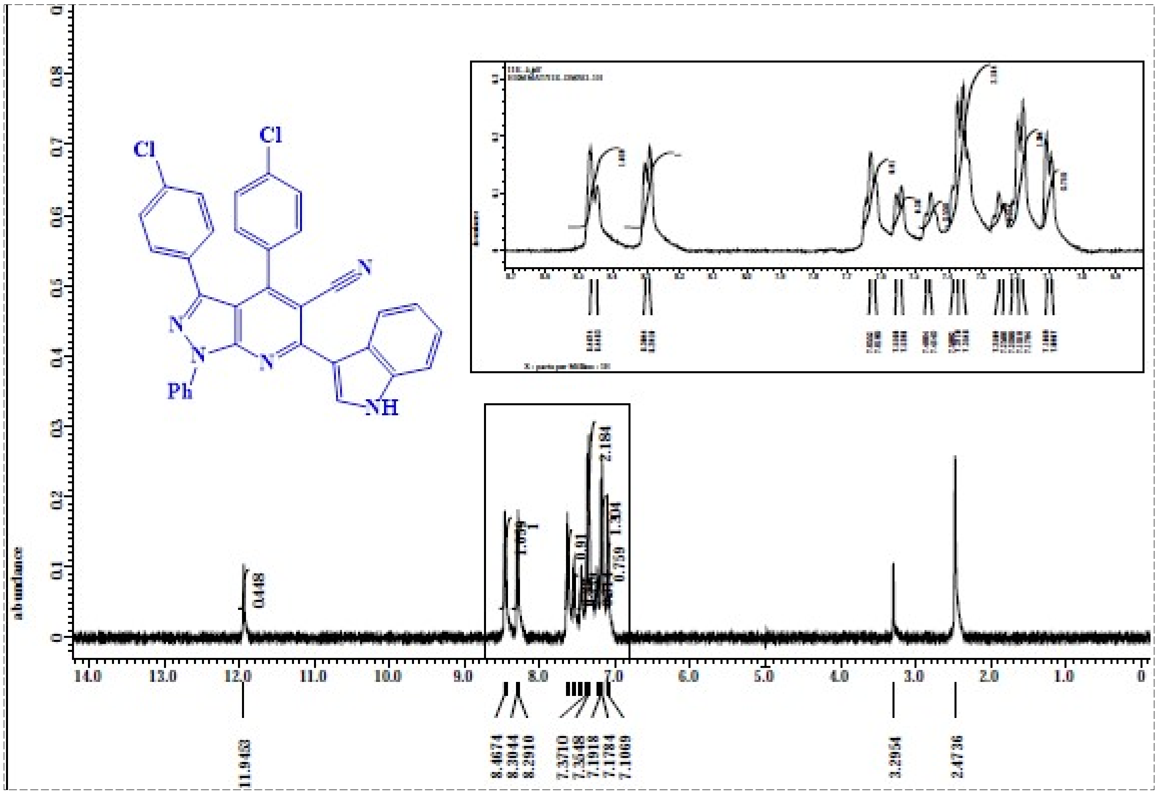

Figure S8. ^{1}H NMR spectrum of compound **4e**

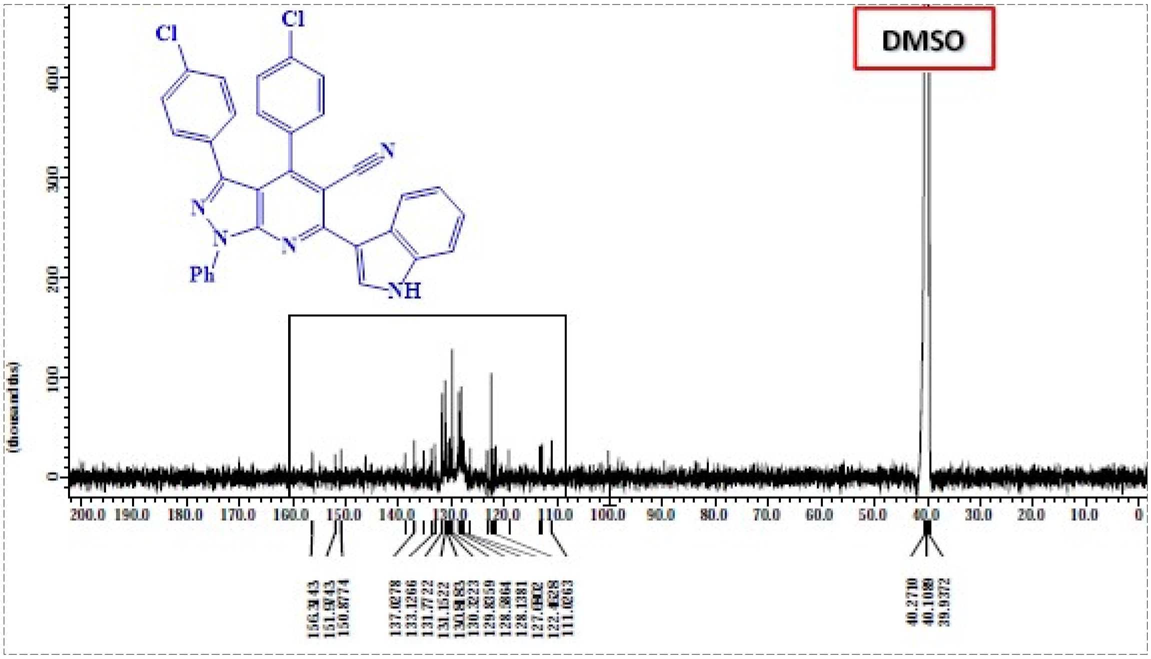

Figure S9. ^{13}C NMR spectrum of compound **4e**

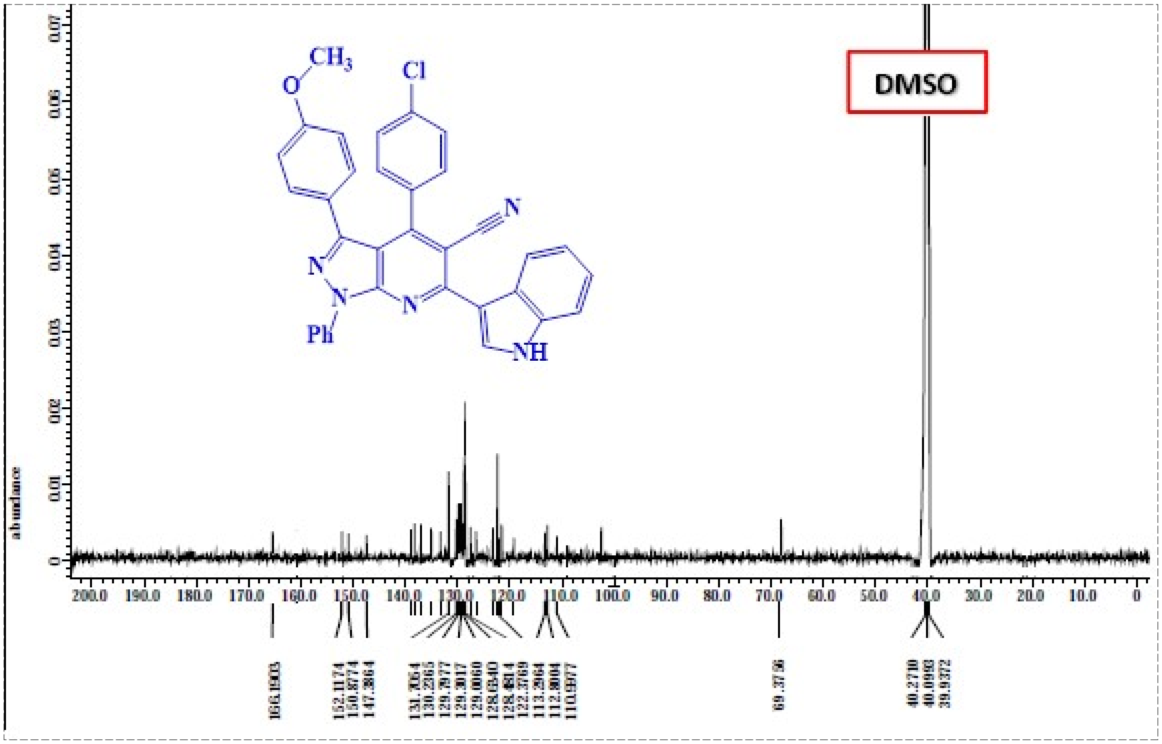

Figure S10. ^{13}C NMR spectrum of compound **4f**

Table S1: Analytical data for the compounds 4a-f

Comp.	Molecular formula (M.Wt)	Color	m/z (%)	Yield (%)	M.P. (^{0}C)	Analysis (calculated)			
						%C	%H	%Cl	%N
**4a**	$C_{34}H_{23}N_5$ (501.59)	Yellow	501 (81)	75	290-291	81.50 (81.42)	4.59 (4.62)		13.91 (13.96)
**4b**	$C_{33}H_{20}ClN_5$ (522.01)	Yellow	521 (20)	77	280-281	76.00 (75.93)	3.84 (3.86)	6.76 (6.79)	13.40 (13.42)
**4c**	$C_{34}H_{23}ClN_5O$ (517.59)	Brown	517 (68)	76	285-286	78.83 (78.90)	4.52 (4.48)		13.59 (13.53)
**4d**	$C_{34}H_{22}ClN_5$ (536.16)	Yellow	535 (88)	77	286-287	76.23 (76.18)	4.09 (4.14)	6.66 (6.61)	13.02 (13.07)
**4e**	$C_{33}H_{19}Cl_2N_5$ (556.45)	Yellow	555 (73)	75	292-293	71.31 (71.23)	3.46 (3.44)	12.69 (12.74)	12.54 (12.59)
**4f**	$C_{34}H_{22}ClN_5O$ (552.03)	Brown	551 (85)	75	295-296	74.06 (73.98)	3.95 (4.02)	6.46 (6.42)	12.64 (12.69)

Table S2: Spectral data for the compounds 4a-f

Comp.	IR (KBr) ν cm^{-1}	^{1}H NMRδ, ppm	^{13}C NMRδ, ppm
**4a**	3377 (NH), 3047 (aromatic H), 2226 (C≡N)	(DMSO-d_6) δ ppm: 2.23 (s, 3H, CH_3), 7.27–7.76 (m, 17H, Ar-H), 8.32 (d, 1H, Ar-H), 8.47 (s, 1H, pyrrole-H), 11.64 (s, 1H, NH, D_2O exchangeable).	(DMSO-d6) δ ppm: 21.2 (CH_3), 111.2-131.8 (C_{Ar}), 147.4 (C-N), 151.2 (C-N), 152.5 (C-N), 157.3 (C-N).
**4b**	3380 (NH), 3045 (aromatic H), 2222 (C≡N)	(DMSO-d_6) δ ppm: 7.26–7.78 (m, 17H, Ar-H), 8.33 (d, 1H, Ar-H), 8.46 (s, 1H, pyrrole-H), 11.75 (s, 1H, NH, D_2O exchangeable).	(DMSO-d_6) δ ppm: 110.8-134.2 (C_{Ar}), 146.1 (C-N), 149.9 (C-N), 151.4 (C-N), 155.1 (C-N).
**4c**	3371(NH), 3050 (aromatic H), 2217 (C≡N)	(DMSO-d_6) δ ppm: 3.68 (s, 3H, OCH_3), 6.58–7.61 (m, 17H, Ar-H), 8.31(m, 1H, Ar-H), 8.46 (s, 1H, pyrrole-H), 11.89 (s, 1H, NH, D_2O exchangeable).	(DMSO-d_6) δ ppm: 65.9 (OCH_3), 110.6-134.9 (C_{Ar}), 148.0 (C-N), 151.6 (C-N), 153.4 (C-N), 166.4 (C-O).
**4d**	3370 (NH), 3055 (aromatic H), 2224 (C≡N)	(DMSO-d_6) δ ppm: 2.25 (s, 3H, CH_3), 6.92–7.63 (m, 16H, Ar-H), 8.32(d, 1H, Ar-H), 8.46 (s, 1H, pyrrole-H), 11.91 (s, 1H, NH, D_2O exchangeable)	(DMSO-d_6) δ ppm: 21.3 (CH_3), 110.9-131.7 (C_{Ar}), 147.3 (C-N), 150.8 (C-N), 152.1 (C-N), 156.1 (C-N).

**4e**	3380 (NH), 3066 (aromatic H), 2219 (C≡N)	(DMSO-d_6) δ ppm: 7.10–7.32 (m, 16H, Ar-H), 8.29(d, 1H, Ar-H), 8.46 (s, 1H, pyrrole-H), 11.94 (s, 1H, NH, D_2O exchangeable).	(DMSO-d_6) δ ppm: 111.0-137.0 (C_{Ar}), 150.8 (C-N), 151.6 (C-N), 156.3 (C-N).
**4f**	3379 (NH), 3082 (aromatic H), 2219 (C≡N)	(DMSO-d_6) δ ppm: 3.70 (s, 3H, OCH_3), 6.90–7.65 (m, 16H, Ar-H), 8.31(d, 1H, Ar-H), 8.45 (s, 1H, pyrrole-H), 11.85 (s, 1H, NH, D_2O exchangeable)	(DMSO-d_6) δ ppm: 69.3 (OCH_3), 110.3-134.7 (C_{Ar}), 147.3 (C-N), 150.8 (C-N), 152.1 (C-N), 166.1 (C-O)
